# Nonlinear Magnetic Response Measurements in Study of Magnetic Nanoparticles Uptake by Mesenchymal Stem Cells

**DOI:** 10.3390/nano15090675

**Published:** 2025-04-29

**Authors:** Vyacheslav Ryzhov, Yaroslav Marchenko, Vladimir Deriglazov, Natalia Yudintceva, Oleg Smirnov, Alexandr Arutyunyan, Tatiana Shtam, Evgenii Ivanov, Stephanie E. Combs, Maxim Shevtsov

**Affiliations:** 1 Petersburg Nuclear Physics Institute Named by B.P. Konstantinov of National Research Centre «Kurchatov Institute», Orlova Roscha 1, 188300 Gatchina, Russia; marchenko_yy@pnpi.nrcki.ru (Y.M.); deriglazov_vv@pnpi.nrcki.ru (V.D.); smirnov_op@pnpi.nrcki.ru (O.S.); arut61@mail.ru (A.A.); shtam_ta@pnpi.nrcki.ru (T.S.); 2 Laboratory of Biomedical Nanotechnologies, Institute of Cytology of the Russian Academy of Sciences (RAS), 194064 St. Petersburg, Russia; yudintceva@mail.ru; 3 LLC “SPF “HELIX”, Sampsonievsky B. Prospect, 20 Litera A, 194044 St. Petersburg, Russia; ivanov61@mail.ru; 4 Department of Radiation Oncology, Klinikum Rechts der Isar, Technical University of Munich, Ismaninger Str. 22, 81675 Munich, Germany; stephanie.combs@tum.de; 5 Personalized Medicine Centre, Almazov National Medical Research Centre, 2 Akkuratova Str., 197341 St. Petersburg, Russia

**Keywords:** magnetic nanoparticles, mesenchymal stem cells, longitudinal response to weak alternating (ac) magnetic field

## Abstract

Stem cells therapies offer a promising approach in translational oncology, as well as in regenerative medicine due to the tropism of these cells to the damage site. To track the distribution of stem cells, the latter could be labeled by MRI-sensitive superparamagnetic (SPM) iron oxide nanoparticles. In the current study, magnetic properties of the magnetic nanoparticles (MNPs) incorporated into the bone marrow-derived fetal mesenchymal stem cells (FetMSCs) were evaluated employing nonlinear magnetic response measurements. Synthesized dextran-coated iron oxide nanoparticles were additionally characterized by X-ray diffraction, transmission electron microscopy, and dynamic light scattering. The MNP uptake by the FetMSCs 24 h following coincubation was studied by longitudinal nonlinear response to weak alternating magnetic field with registration of the second harmonic of magnetization. Subsequent data processing using a formalism based on the numerical solution of the Fokker–Planck kinetic equation allowed us to determine magnetic and dynamic parameters and the state of MNPs in the cells, as well as in the culture medium. It was found that MNPs formed aggregates in the culture medium; they were absorbed by the cells during coincubation. The aggregates exhibited SPM regime in the medium, and the parameters of the MNP aggregates remained virtually unchanged in the cells, indicating the preservation of the aggregation state of MNPs inside the cells. This implies also the preservation of the organic shell of the nanoparticles inside FetMSCs. The accumulation of MNPs by mesenchymal stem cells gradually increased with the concentration of MNPs. Thus, the study confirmed that the labeling of MSCs with MNPs is an effective method for subsequent cell tracking as incorporated nanoparticles retain their magnetic properties.

## 1. Introduction

The search for new approaches for targeted delivery of diagnostic and therapeutic agents is one of the priority areas in modern translational and practical oncology [1,2]. In addition to various biological molecules (e.g., peptides, proteins, monoclonal antibodies, nanobodies, etc.) and nanosize agents (magnetic nanoparticles, quantum dots, magnetosomes, etc.), the use of cellular drug-delivery systems deserves special attention [3,4]. Promising candidates for cellular delivery systems include immune cells (e.g., neutrophils, macrophages, T lymphocytes), blood cells (particularly erythrocytes, platelets), and stem cells, which have been shown in numerous studies to have an innate tropism for tumor tissue and low immunogenicity [5,6,7,8,9,10,11]. This cell-based delivery system as compared to conventional approaches additionally was shown to exhibit prolonged circulation, low cytotoxicity profile, escape to clearance by the reticuloendothelial system, high biocompatibility, and good biodegradable properties which, in turn, results in highly efficient delivery of theranostic agents to the tumor site with a reduction of side effects due to the decrease of the off-site drug effects [12,13]. It is worth emphasizing separately that cellular delivery systems also make it possible to effectively bypass various histo-hematic barriers (e.g., blood-brain barrier), which, as in the case of glioblastoma multiforme, can significantly complicate the delivery of drugs [14]. Indeed, several studies demonstrated retention of cell-based systems in the glioma tissues passing the BBB, including the RBC-based [15], dendritic cell-based [16], and neutrophil-based systems [17]. The tropism of stem cells of various origins (widely used mesenchymal, as well as hematopoietic and embryonic) to tumors has also been shown in preclinical models of glioblastoma [18]. Apart from the inherent tumor tropism, which facilitates the drug delivery, a cell-based system might also exert an additional anti-tumor effect, as was shown for the immunomodulatory effect of the stem-cell-based delivery system [12,19,20].

Mesenchymal stem cells (MSCs) are easily isolated, cultured, and manipulated ex vivo. They have the potential to produce progeny that differentiate into a variety of cell types, showing great potential for therapeutic applications [21]. Currently, MSCs (either derived from bone marrow, cord blood, or adipose tissue) are already used in preclinical and clinical trials, and MSC-based drug delivery can be considered as a promising new approach to tumor theranostics [22]. The applying of magnetic nanoparticles (MNPs) in tumor theranostics, on the one hand, expands the possibilities of early magnetic resonance imaging (MRI) diagnostics [23] and, on the other hand, makes it possible to use radiosensitization [24,25,26,27] and hyperthermia [28,29,30] for tumor therapy. MNPs as magnetic labels also enable for the testing of the efficiency of targeted delivery of therapeutic agents by applying a unique technique of longitudinal nonlinear response to alternating magnetic field with the registration of the second harmonic of magnetization (NLR-M2) developed before [31]. Indeed, several studies have shown the possibility of tracking stem cells loaded with magnetic particles in the diagnosis of pathological processes in the body [32,33,34,35,36]. Presumably, hydrogels can also be used to deliver MNPs [37]. Apart from cell tracking and drug delivery, the MNP-loaded MSCs have demonstrated efficacy in tumor treatment and tissue regeneration [38,39,40,41].

The use of MSC tropism for the delivery of MNPs (they may be conjugated with therapeutic agents) to the sites of inflammation requires quantitative studies of their uptake by the cells and possible transformation of their state inside the cells under the influence of various cellular systems. The latter can be controlled by assessing the preservation/change of magnetic and dynamic properties of the magnetic centers producing the NLR-M2 signal. Such information is important for the estimation of the potential of MSCs for targeted delivery and is practically absent in the literature. In this work, the dextran-coated magnetite nanoparticle incorporation into MSCs during coincubation at various Fe concentrations was studied. Magnetic and dynamic parameters of MNPs inside the cells and in the culture medium were determined to clarify possible changes in their state, and their possible toxic effect on the cells was tested.

## 2. Materials and Methods

### 2.1. Synthesis of Iron Oxide Nanoparticles Coated by Dextran Shell

Iron oxide nanoparticles were synthesized in the same way as described previously [42,43]. Co-precipitation of iron salts (III) and (II) in the ratio 2:1 in an alkaline medium was applied. An aqueous solution of ammonia was used as a precipitant. Nanoparticles were stabilized with dextran (9–11 kDa) using ultrasound. To remove large aggregates, the resulting suspensions were kept for 24 h in the magnetic field of a permanent magnet with an induction of 1.3 T, and the supernatant was collected. The resulting colloidal solutions of MNPs were stored at a temperature of 4 °C.

### 2.2. Study of the Structure of Iron Oxide Nanoparticles by X-Ray Diffraction

The composition and structure of the synthesized MNPs were examined by XRD. The DRON-3M diffractometer (Joint Stock Company “Innovation Center “Burevestnik”, Gatchina, Russia) was used, providing the Cu K_α_ radiation line with the wavelength λ = 1.5405 Å. The size of the MNP crystallinity region was evaluated by precise treatment of the XRD pattern with the account of instrumental resolution and a doublet structure of the Cu K_α_ line.

### 2.3. Transmission Electron Microscopy

A drop of the solution was placed on a glass substrate, excess water was removed with filter paper, and the wet sample was immediately examined with the electron microscope JEM-100C (Jeol, Tokyo, Japan).

### 2.4. Dynamic Light Scattering

The DLS measurements were performed at room temperature using the Photocor Compact-Z device manufactured by LLC Fotokor (Moscow, Russia). The radiation source was a stabilized laser photodiode with a light wavelength of 637.7 nm, providing a maximum output radiation power of 25 mW. The scattering angle was 90°. The scattered light was registered by a highly sensitive photon-counting system based on an avalanche photodiode with a single-mode light guide at the input (Excelitas Technologies, Pittsburgh, PA, USA) equipped with a thermoelectric cooling system. The built-in Photocor-FC correlator calculated and accumulated the correlation function of intensity fluctuations of the scattered light in real time. A glass cuvette was used for the sample, and its holder was thermostatically controlled. The size distributions were obtained from processing the scattered light correlation function by the regularization method built into the instrument software as a separate code. The mass distribution of particles calculated from the light scattering intensity was recalculated into the size distribution, assuming that the scattering particles are balls of the same material.

### 2.5. Determination of MNP Concentration in Suspension

The concentration of iron in the colloidal aqueous solutions of MNPs was measured by the thiocyanate method based on the colorimetric determination of the formation of a colored iron thiocyanate complex during the interaction of iron (III) ions with potassium thiocyanate [44]. For this purpose, MNPs were converted into the ionic form of Fe^3+^ by exposing Fe_3_O_4_ nanoparticles to concentrated nitric acid while heated at 80 °C for 10 min in a water bath. The obtained solution was chilled to room temperature, diluted with distilled water, and mixed with the 0.8 M aqueous solution of potassium thiocyanate. A minute after the start of the complexation reaction, the optical density of the resulting solution was measured at a wavelength of 575 nm against the control. The measurements were conducted on the Genesys 50 spectrophotometer (Thermo Fisher Scientific Inc., Waltham, MA, USA). The iron concentration of the samples under study was determined from the calibration graph composed using a number of standard solutions of ferroammonium alum.

### 2.6. MSC Culture

Human fetal mesenchymal stem cells derived from bone marrow (FetMSCs) were obtained from the shared research facility “Vertebrate Cell Culture Collection” supported by the Ministry of Science and Higher Education of the Russian Federation at the Institute of Cytology of the Russian Academy of Sciences (St. Petersburg, Russia). FetMSCs were harvested in the DMEM culture medium (Sigma-Aldrich, St. Louis, MO, USA) supplemented with 10% fetal bovine serum, 100 U/mL penicillin, and 100 µg/mL streptomycin (Gibco, Waltham, MA, USA) in a CO_2_ incubator (Thermo Fisher Scientific, Waltham, MA, USA) at 37 °C with 5% carbon dioxide, with regular changes of the culture medium.

### 2.7. Coincubation of MSCs with MNPs

The cells were incubated with PBS (phosphate-buffered saline) (control) and MNPs (at Fe concentrations of 50, 100, 150, and 300 μg/mL) for 24 h in a CO_2_ incubator. Following incubation, the cells were washed and their viability was assessed by 0.4% Trypan blue exclusion. Additionally, a 3-(4,5-dimethylthiazol-2-yl)-2,5-diphenyl tetrazolium bromide (MTT) assay was used to test cytotoxicity of the MNPs and the cell viability. Vybrant^®^ MTT Cell Proliferation Assay Kit was applied according to the manufacturer’s protocol (Life Technologies, Waltham, MA, USA). Then, the samples for NLR-M2 measurements were prepared: (i) the culture medium with dispersed MNPs before the coincubation with the cells; (ii) the cells with absorbed MNPs washed from the residual medium and MNPs with the phosphate buffer after the coincubation and resuspended in PBS. The number of cells was counted using the Countess II FL Automated Cell Counter (Thermo Fisher Scientific, Waltham, MA, USA). The experiments were conducted in five replicates.

### 2.8. NLR-M2 Measurements

To determine the magnetic and dynamic parameters of nanoparticles in the culture medium and in the cells after cultivation, a highly sensitive method of longitudinal nonlinear response to the weak ac magnetic field *h*(*t*) parallel to the constant field *H* was applied. Based on this method, a previously described [45] homemade installation was adapted for studying MNPs [46]. The dependences of the phase components of the second harmonic of magnetization, namely, the real and imaginary parts Re*M*_2_ and Im*M*_2_ of the signal on the steady magnetic field *H* were simultaneously recorded. The experiments were conducted at room temperature while stabilizing the sample temperature by a flow thermostat with evaporated nitrogen as a coolant. The field *H* was slowly scanned in the range from −300 to 300 Oe and backwards symmetrically to the point *H* = 0. Two scan frequencies, *F*_sc_ = 0.25 and 8 Hz, were enabled to control the field hysteresis in the signal. The scan frequency dependence of the hysteresis would mean its dynamical character, which, in turn, would evidence SPM nature of the magnetic centers [47]. The sensitivity of the setup was about 10^−10^ emu.

### 2.9. Processing Nonlinear Response Data

For quantitative assessment of magnetic and dynamic parameters of the studied MNPs, the obtained Re*M*_2_(*H*) and Im*M*_2_(*H*) dependences were processed using a formalism based on the numerical solution of the Fokker–Planck kinetic equation for SPM particles as it had been done previously at studying the state of the dextran-coated MNPs in a colloidal aqueous solution [47].

The magnetic potential of SPM MNPs with uniaxial anisotropy has two minima corresponding to two possible magnetization directions. Permanent magnetic field tends to magnetize the MNPs along the field. Reversing the field changes the magnetization direction for the opposite one. Due to the potential barrier separating the two minima, the magnetization retards from the field (Neel relaxation). This time lag depends on the relationship between the field reversal rate and the longitudinal relaxation time, the intrinsic parameter of MNPs. Hence, there is periodic scanning of the magnetic field, resulting in a field hysteresis of magnetization. The shorter the scanning period, the less time the MNP ensemble has to relax to the equilibrium state, resulting in a wider hysteresis observed. The dependence of the hysteresis width on the scan frequency of the *H* field is indicative of the SPM character of MNPs. Another reason for hysteresis can be viscous friction of magnetic objects when they rotate in the solution under reversal of *H* field (Brownian relaxation). An absence or only a small magnetic hysteresis justifies the application of the FP formalism.

The computational resources of PIK Data Processing Centre of NRC “Kurchatov Institute”—PNPI (Gatchina, Russia) were exploited with the in-house provided software. As required by the model, the raw data were averaged between the forward and backward scans and antisymmetrized about *H* = 0 before the parameter-fitting procedure. A number of parameters largely characterizing the MNP ensembles in the samples under study was obtained such as the lognormal distribution width *σ* and the mean value of magnetic moments *M*_C_, the saturation magnetization *M*_S_, the concentration of magnetic centers *N*_C_ proportional to the saturation magnetization, and the magnetic anisotropy, as well as the parameters of magnetization dynamics like the damping factor *α* and the longitudinal relaxation time *τ_N_*.

If the signal-to-noise ratio of the *M*_2_ response is poor, noticeable errors can appear in the parameters obtained. As the condition *M*_2_ ∝ *h*^2^ is satisfied, the moduli of the integral *M*_2_ signals normalized to *h*^2^ can be used for the assessment of the relative content of the magnetic centers in different samples with similar magnetic moment distributions. In particular, one can estimate the content of MNPs absorbed by the cells at coincubation, comparing their *M*_2_ signals with that of the medium before coincubation with the known concentration of Fe [48]. This approach allows one to obtain a more reliable estimate of the concentration of magnetic centers in the samples with small Fe contents.

### 2.10. Statistics

The numerical results were collected in a table and statistically processed using GraphPad Prism 9 software (GraphPad Software Inc., Boston, MA, USA). The results of the study were expressed as the mean (M) ± standard deviation (SD). The statistical comparisons between two groups of parameters were performed using unpaired *t*-tests, and the comparisons between more than two groups were conducted employing one-way analysis of variance (ANOVA); *p*-value less than 0.05 was considered statistically significant.

## 3. Experimental Results

### 3.1. Characterization of the MNPs

The study of MNP incorporation into mesenchymal stem cells required the nanoparticles to detail characterization. This included identification of the substance and determination of the crystal structure and the crystallinity region of the MNP magnetic core by XRD. The state of the MNP ensemble in a colloidal solution was examined by TEM and DLS. Attestation results enable for the correct interpretation of magnetic and dynamic parameters of the MNP ensemble in the suspension.

#### 3.1.1. Structure of Iron Oxide Nanoparticles from X-Ray Diffraction

**Figure 1:**
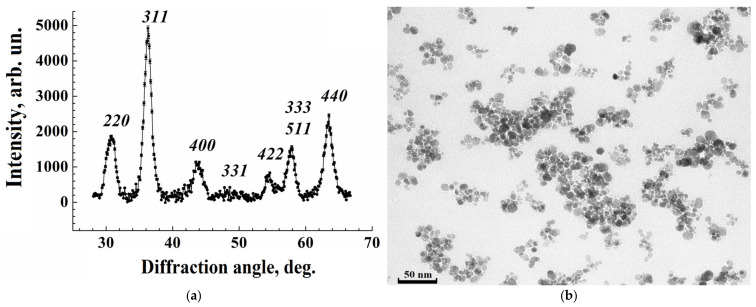
X-ray diffraction intensity from MNPs vs. diffraction angle (**a**) and TEM image of nanoparticles (**b**).

The MNP structure, composition, and crystallite size were examined by XRD with continuous scanning in θ–2θ configuration in the range 25°–70°. The XRD intensity dependence on the diffraction angle registered at room temperature is represented in Figure 1a. All Bragg reflections are indexed, confirming the formation of the well-defined single-phase cubic spinel Fe_3_O_4_ structure with the lattice parameter 0.8397 nm without any impurity peaks. Fundamental *hkl* reflections from the crystal planes (220), (311), (400), (422), (511), and (440), characterizing the spinel ferrites, were clearly identified. The broadening of the diffraction peaks arises from a finite size of the coherent scattering region and internal stress in the sample. The Williamson–Hall approach was applied to differentiate between the size- and strain-induced broadenings [49]. The average size of the crystallinity area was found to be 9.3 (6) nm, while the strain-induced broadening was small, as in the previous results.

#### 3.1.2. Examination by Transmission Electron Microscopy

A fragment of the TEM micrograph from a drop of the aqueous colloidal solution of iron oxide MNPs in a dextran shell on a glass substrate is represented in Figure 1b. The micrograph was made on the damp sample after carefully removing excess water with filter paper, so that the aggregation state of MNPs did not change. From the figure, the nanoparticles have a shape close to a sphere with a diameter of ~10 nm and form aggregates of irregular shape and different sizes in agreement with previous results [47]. The distribution of aggregate sizes as a function of the diameter of the effective sphere approximating the aggregate is well described by the lognormal distribution with the average value of 44 nm. The contrast of the micrograph is provided by the magnetite cores, and the dextran shells around the cores are only slightly visible as thin gaps ~1.5 nm between dark spots. The average diameter of the cores is 9.46 (15) nm, within the measurement accuracy, which corresponds to the crystallinity size found from XRD. This indicates the absence of a non-crystalline phase in the magnetite fraction. Numerous experimental and theoretical estimates show magnetite particles of ~10 nm in size to be in a single-domain state. The threshold of this state is estimated to be from 20 to 130 nm, depending on the technological conditions of their manufacturing [50].

From TEM and XRD indicating single-crystal magnetite nanoparticles with an average volume of 688 nm^3^ over the volume distribution, the MNP average magnetic moment was estimated to be 2.8∙10^4^ μ_B_.

#### 3.1.3. Dynamic Light Scattering Results

**Figure 2:**
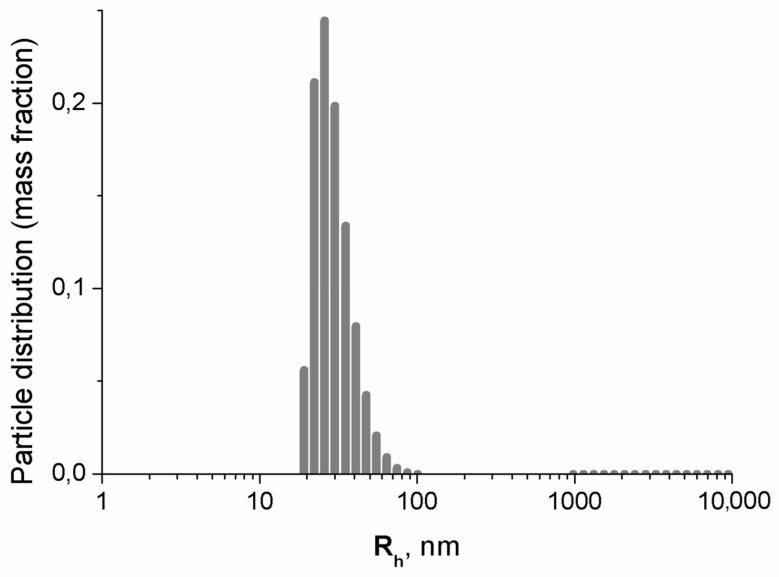
Distribution of hydrodynamic radii of MNPs’ aggregates in colloidal aqueous solution (histogram).

The mass distribution of hydrodynamic radii of colloidal particles in the suspension is displayed in Figure 2. The average diameter of 60 (6) nm coincides within the error bars, with the average diameter 52 (7) nm of the aggregates obtained by DLS in [47] for freshly synthesized MNPs after centrifugation (g = 10,000, t = 5 min). No heavier fraction was observed after the magnet treatment.

### 3.2. Evaluation of the Toxicity of Nanoparticles

The cell viability, as assessed by the trypan blue exclusion assay, did not vary during the incubation periods (1, 3, and 12 h), with MNPs at concentrations of 50, 100, 150, and 300 µg/mL and did not differ from the control cells (*p* ≤ 0.05), which is consistent with the previous results [51]. After 24 h of cell coincubation with MNPs at concentrations of 50, 100, and 150 μg/mL, no toxic effect was observed, and only a slight decrease of cell viability (about 10%) was detected at the maximal MNP concentration of 300 μg/mL. The standard MTT assay did not reveal any influence of MNPs on cell viability; it did not differ from the control (Figure S1).

### 3.3. NLR-M2 Results

**Figure 3:**
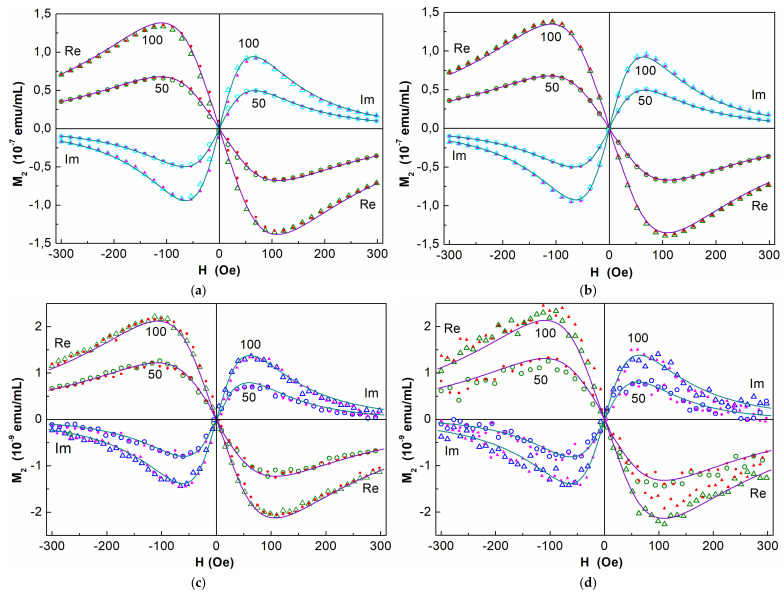
Real and imaginary parts of *M*_2_ response as functions of dc magnetic field from suspensions of MNPs in medium before coincubation with MSCs (**a**,**b**), and from MSCs washed with phosphate buffer after coincubation (**c**,**d**), at iron concentrations of 50 and 100 μg/mL. The signals were registered at *H*-scan frequencies 8 Hz (**a**,**c**) and 0.25 Hz (**b**,**d**), respectively. Filled and open symbols present direct and reverse *H* scans, accordingly, and solid curves display simultaneous best fit of both phase components for each concentration. Every 60th point is shown in (**a**) and (**b**), and every 40th point in (**c**) and (**d**) panels, respectively.

Figure 3 represents the phase components of the *M*_2_ response from colloidal suspensions of MNPs in the incubation medium before co-incubation with cells, recorded at room temperature, an ac-field amplitude of 4.7 Oe, *F_scan_* = 8 Hz (panel (a)), and 0.25 Hz (panel (b)). Comparison of these signals shows that an increase in the steady field scan period and, accordingly, the time allotted for the relaxation of the MNP ensemble from 0.125 to 4 s is accompanied by the disappearance of the field hysteresis. This indicates the SPM mode of the MNP ensemble and the establishment of an equilibrium state quicker than in 4 s per scan cycle (2 ms per each *H* point). The *M*_2_ response of the cells with absorbed MNPs (panels (c) and (d)) is two orders of magnitude weaker than that of the incubation medium with MNPs because the cells ingest only a small fraction (~1%) of the particles from the medium. Since noise masks the field hysteresis of the response, it is difficult to evaluate it at a scan rate of 0.25 Hz. However, close values of the parameters obtained for the cells with MNPs and for the medium with MNPs before incubation (see Table 1) together with only slight *H* hysteresises at *F*_sc_ = 8 Hz (panels (a) and (c)) with a good signal-to-noise ratio verify the SPM mode of the MNP ensemble in both systems [47] and justify the FP formalism used.

Similar forms of Re*M*_2_(*H*) and Im*M*_2_(*H*) signals and close ratios of the amplitudes of the phase components from the medium before incubation and from the cells with incorporated nanoparticles after coincubation suggest a virtually identical state of the aggregates of MNPs in the medium and in the cells after incorporation. This is also confirmed by the close values of their magnetic parameters (Table 1).

In Table 1, all parameters match within the error limits. Magnetic anisotropy energy in all cases was found to be 10–20 K. The Néel relaxation time 1–2 ns is typical for SPM mode in agreement with the above discussion and the previously obtained results [47,48], as well as the width of the lognormal distribution of the moments of magnetic centers *ϭ* ~ 0.7. To characterize the MNP content in the medium for incubation and the efficacy of their incorporation into the cells, we use further, mainly, the concentration of magnetic centers *N*_C_ and their mean magnetic moment *M*_C_. Aggregation minimizes the magnetostatic energy of the MNP ensemble and, accordingly, its free energy, as it was found earlier [47]. The magnetic dipole–dipole coupling between MNPs in the aggregate is rather strong. The characteristic dipolar energy corresponds to the field 1.4 kOe [47], much larger than the range of the dc field used in the measurements (±300 Oe). Therefore, the magnetic response is a collective effect of all MNPs constituting the aggregate, and the magnetic centers are associated with the aggregates rather than with separate MNPs. The moduli of the *M*_2_ signal integrals normalized to *h*^2^ can be used for the assessment of the relative content of the magnetic centers in different samples with similar magnetic moment distributions.

The mass of the MNP magnetite core with an average core size of ~9 nm is *m_Fe_* ≈ 2.59·10^−9^ ng/MNP [47]. Thus, with the known concentration of Fe in the suspension, we can estimate the average concentration of MNPs *N*_P_ in the medium. The ratio *N*_ag_ = *N*_P_/*N*_C_ allows one to determine the average amount of MNPs per aggregate for all concentrations used. However, a poor signal-to-noise ratio in the *M*_2_ response from the cell suspension after incubation indicates a small relative amount of absorbed MNPs, and the thiocyanate method does not allow for a reliable assessment of the amount of iron contained in the cells against the background of metal ions in them. Therefore, the iron content in the cells was estimated using the ratio of the integrals of the signals from the washed cells after incubation and from the medium with MNPs before incubation with the known content of iron in the latter.

**Figure 4:**
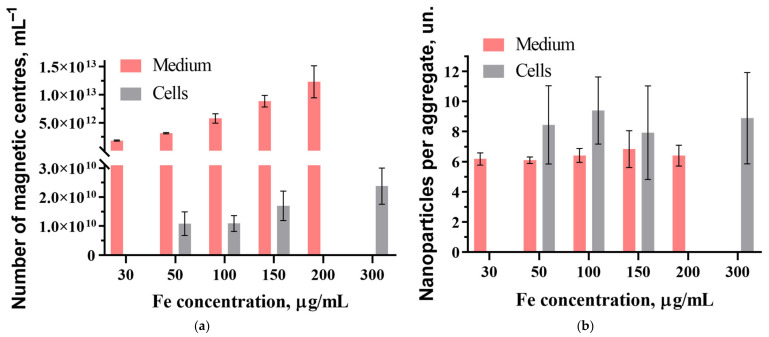
Average numbers of magnetic centers (aggregates) (**a**) and MNPs per aggregate (**b**) in medium before coincubation of MSCs with MNPs and in the samples with the cells washed with the phosphate buffer after coincubation at different concentrations of MNPs.

The obtained average values of *N*_C_ and *N*_ag_ in the medium before incubation and in the samples with the cells washed with PBS after coincubation with MNPs at different concentrations are shown in Figure 4.

From Figure 4a, the average concentration of aggregates absorbed by the cells *Nc* increases monotonically with the MNP concentration in the medium indicating the absence of saturation of the MNP uptake. The average numbers of MNPs per aggregate in MSCs and in the medium coincide within the error limits, as Figure 4b shows, indicating the preservation of the aggregate state of MNPs in the cells and conservation of the dextran shell of the nanoparticles.

**Figure 5:**
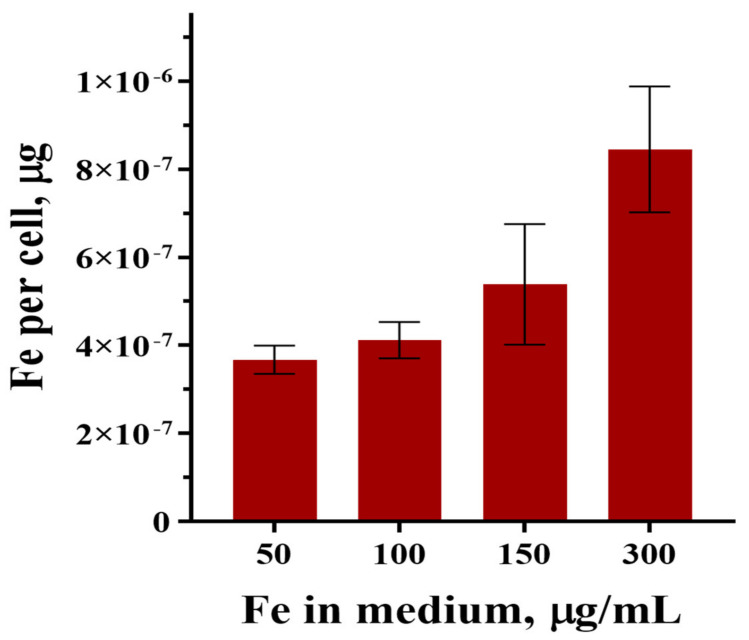
The average number of MNPs per MSC after 24 h of coincubation at different Fe concentrations.

Before recording the *M*_2_ response from the samples, the number of cells was counted to be ~6 × 10^5^ 1/mL, which made it possible to estimate the average number of MNPs absorbed by a cell during coincubation. Figure 5 represents the amount of iron per cell after 24 h coincubation in the media with the contents of MNPs corresponding to *C*_Fe_ = 50, 100, 150, and 300 μg/mL.

From Figure 5, the amount of iron in the MNPs absorbed by the cells during coincubation increases with the iron concentration. The known mass of iron contained in the magnetite core of dextran-coated nanoparticles synthesized by the same method [47] allows us to estimate the average number of MNPs absorbed by one cell after 24 h of coincubation as ~1.6 × 10^5^ and ~3.3 × 10^5^ 1/mL at *C*_Fe_ = 100 and 300 μg/mL, respectively.

## 4. Discussion

In light of the application of stem cell-based technologies for diagnostics and therapy of various diseases (including tumors, degenerative diseases, etc.), the study of the biodistribution of the introduced cells is of particular interest. In this regard, the development of stable labels for cell labeling has developed significantly in recent decades [52]. Along with existing fluorescent dyes and gene-reporter structures, the use of synthetic magnetic nanoparticles (based on magnetite) is of particular interest due to the relative simplicity of obtaining the particles and low costs, low cytotoxicity, the presence of special physicochemical properties that allow for the detection of nanoparticles using MRI with high spatial resolution [53,54,55,56,57]. In this study, dextran-coated iron oxide nanoparticles were used to label FetMSCs. Considering the presence of a bioorganic coating of the particles and their preferential penetration into the cell via the endolysosomal pathway (with subsequent degradation under the influence of aggressive enzymes in endolysosomes), the question remains open as to how long the particles can retain their diagnostic potential (i.e., magnetic properties determined by MRI examination) when penetrating into the cell [58,59]. Unlike previously presented methods, this work used the nonlinear magnetic response method, which allowed us to estimate the magnetic properties of the particles with a high degree of reliability, as well as to determine in what state (single- or multidomain) they are in endolysosomes and in the cells, which, in turn, allowed us to draw a conclusion about their functional activity. The multidomain state is formed in the cells after the destruction of the organic shell of nanoparticles, accompanied by the adhesion of their magnetic cores, and is characterized by the presence of field hysteresis and a decrease of the signal amplitude in the *M*_2_ response. Then, the magnetite core of MNPs is destroyed by the cells. Such a picture was observed in kidney tissue while studying the biodistribution of MNPs in a preclinical model of renal tuberculosis [31].

As was established in the present work, the amount of iron absorbed by a cell and, accordingly, the amount of absorbed MNPs during coincubation (Figure 5) grows with increasing iron concentration without changing their magnetic and dynamic parameters (Table 1). The values of the obtained parameters are close to the parameters of MNP aggregates in the medium before coincubation. This means that the cells absorb aggregates from the medium without their destruction. The monotonic growth of MNPs absorbed by the cells with increasing MNP concentration in the incubation medium indicates the absence of saturation in the concentration range under consideration, as was observed earlier while studying the absorption of MNPs by glioblastoma cells of A172 and Gl-Tr lines, as well as the cells of normal morphology and human embryonic lung fibroblasts (FLEH) [48]. The average diameters of the magnetic cores of MNPs are consistent with the previously obtained result for dextran-containing nanoparticles synthesized by the method used in the present work [47], indicating good reproducibility of the synthesis.

The distribution of aggregate sizes in the TEM micrograph as a function of the diameter of the effective sphere approximating the aggregate was found to be well described by the lognormal distribution with the average value 44 nm, whereas the average diameter of the aggregates according to DLS measurements was determined to be 60 (6) nm in agreement with the previous results [47]. The reason for the difference between the TEM and DLS values is that the hydrophilic dextran shell of the particles couples with water, creating a solvent layer bound to the aggregate surface. A portion of water can also penetrate into the aggregate. Moreover, due to their irregular form, the aggregates carry along an additional amount of water when moving. This bound water increases the effective mass of MNP along with the measured hydrodynamic radius.

The average number of MNPs absorbed by one cell after 24 h of coincubation at *C*_Fe_ = 100 μg/mL was found to be ~1.6 × 10^5^. For comparison, the same quantity for the cells of normal morphology, human embryonic lung fibroblasts (FLEH), is ~1.8 × 10^5^, and for the cells of glioblastoma A172, it is ~2.1 × 10^6^ [48]. This assessment shows that under identical incubation conditions, mesenchymal stem cells absorb significantly less dextran-coated nanoparticles than glioblastoma cells and approximately the same as FLECH cells of normal morphology. This probably suggests the restructuring of the cancer cell membrane, accompanied by a significantly higher permeability, probably to provide nutrition for rapid growth. It may be due to an increase in the number of non-specific channels in the membrane, which provides more efficient absorption of nutrients from the environment. Another explanation for the observed differences in nanoparticle uptake could be explained by differing phagocytic capacities in different cell cultures. Presumably, in FetMSCs, the level of phagocytic activity is less than in previously reported glioblastoma cells [48]. Indeed, as was shown in the study by Costela Ruiz et al. [60], within three human adipose tissues MSC (HAT-MSC), the phagocytic capacity ranged from 33.8% and 56.2% [60]. One of the approaches to increase the uptake of nanoparticles by mesenchymal stem cells and, therefore, increase their diagnostic potential (for example, when conducting MRI studies), could be functionalization of the particle surface with active bioligands (peptides, antibodies, etc.). Indeed, several studies have demonstrated that decoration of the nanoparticle surface with MSC-targeting molecules might significantly increase the cellular incorporation of particles [61,62].

## 5. Conclusions

The nanoparticles absorbed by MSCs during coincubation retain their aggregated state and SPM behavior, their dextran shell is not destroyed, and their magnetic and dynamic parameters have values close to those of MNP aggregates in the culture medium. Thus, dextran-coated magnetite nanoparticles can potentially be used for loading into mesenchymal stem cells. The tropism of MSCs suggests the possibility of targeted delivery of nanoparticles to malignant neoplasms. The latter will increase the efficiency of early MRI diagnostics and allow for the use of tumor therapy based on the physical effects of radiosensitization and hyperthermia of MNPs.

## Figures and Tables

**Table 1:** Parameters of MNPs’ aggregates in medium before incubation with MSCs and in cells after incubation for iron concentrations of MNPs 50 and 100 µg/mL. The parameters are averaged over five measurements and two scan frequencies *F_sc_* = 0.25 and 8 Hz.

Type of Sample	Medium with MNPs Before Incubation				Cells with MNPs After Incubation			
C_Fe_ of MNPs, μg/mL	M_center_, µ_B_	σ	α	τ_N_, ns	M_center_, µ_B_	σ	α	τ_N_, ns
50	3.5(2)∙10^4^	0.65(1)	0.18(1)	1.26(3)	3.0(2)·10^4^	0.71(4)	0.17(2)	1.15(11)
100	3.8(4)·10^4^	0.6(1)	0.18(3)	1.49(26)	3.4(3)·10^4^	0.67(4)	0.18(1)	1.23(14)

## Data Availability

The data presented in this study are available on request from the corresponding author.

## Supplementary Materials

The following supporting information can be downloaded at: https://www.mdpi.com/article/10.3390/nano15090675/s1, Figure S1: MTT assay for cells coincubated with SPIONs at the concentration 300 µg/mL in 24 h.

## Abbreviations

The following abbreviations are used in this manuscript:

MNP	Magnetic nanoparticle
MSC	Mesenchymal stem cell
SPM	Superparamagnetic
NLR-M2	Nonlinear longitudinal response to weak ac magnetic fields with registration of the second harmonic of magnetization
TEM	Transmission electron microscopy
ac	Alternating current
dc	Direct current
DLS	Dynamic light scattering
XRD	X-ray diffraction
MRI	Magnetic resonance imaging
