# Supplementary material for: Nonlinear Magnetic Response Measurements in Study of Magnetic Nanoparticles Uptake by Mesenchymal Stem Cells
Source: Nanomaterials (Basel). 2025 Apr 29;15(9):675. doi: 10.3390/nano15090675 (PMC12073591; doi:10.3390/nano15090675)
Supplement: Supplementary file 1 [file nanomaterials-15-00675-s001.zip › nanomaterials-3506305-supplementary.pdf]

Supplementary information

Nonlinear Magnetic Response Measurements in Study of Magnetic Nanoparticles Uptake by Mesenchymal Stem Cells

Vyacheslav Ryzhov [1,*], Yaroslav Marchenko [1], Vladimir Deriglazov [1], Natalia Yudintceva [2], Oleg Smirnov [1], Alexandr Arutyunyan [1], Tatiana Shtam [1], Evgenii Ivanov [3], Stephanie E. Combs [4] and Maxim Shevtsov [2,4,5,*]

[1] Petersburg Nuclear Physics Institute Named by B.P. Konstantinov of National Research Centre «Kurchatov Institute», Orlova Roscha 1, 188300 Gatchina, Russia
[2] Laboratory of Biomedical Nanotechnologies, Institute of Cytology of the Russian Academy of Sciences (RAS), 194064 St. Petersburg, Russia
[3] LLC "SPF "HELIX", Sampsonievsky B. Prospect, 20 Litera A, 194044 St. Petersburg, Russia
[4] Department of Radiation Oncology, Klinikumrechts der Isar, Technical University of Munich, Ismaninger Str. 22, 81675 Munich, Germany
[5] Personalized Medicine Centre, Almazov National Medical Research Centre, 2 Akkuratova Str., 197341 St. Petersburg, Russia
* Correspondence: ryzhov_va@pnpi.nrcki.ru (V.R.); maxim.shevtsov@tum.de (M.S.)

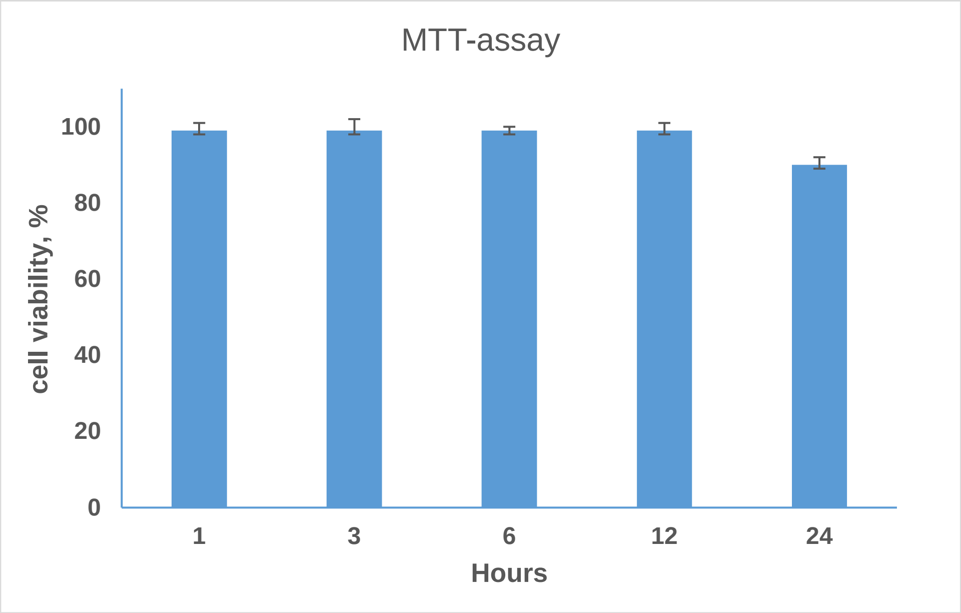

Figure S1. MTT assay for cells coincubated with SPIONs at the concentration 300 µg/mL in 24 hours.
